# Median Lithotomy

**Published:** 1884-09

**Authors:** 


### MEDIAN LITHOTOMY.

This operation was performed by Dr. Wm. L. Axford, who verbally cited the history of the following case of genito-urinary trouble. An old man, ætat 66, of inherited gouty diathesis, gardener,had been during the last year and a half a sufferer from repeated attacks of nephralgia, which could only be relieved by hypodermics of morphia sulphas gr. ¼. After several attacks this remedy seemed to have no effect in affording him relief, neither did suppositories of belladonna and other anodynes appear to have any control over the pain. He was also a sufferer from chronic cystitis. Some time since, the patient was again seized with the kidney trouble and cystic, combined, which continued three days; his urine was voided in small quantities, very often, as frequently as every five minutes, part of the time. The bladder was full continuously, and catheterization was resorted to every six hours. This produced excessive pain, which continued in spite of large doses of opium, for four hours afterwards. Hyperæsthesia of the uretha became very marked, and the procedure of using the catheter had to be abandoned. It was also ascertained that he had an enlarged prostate. As a last resort the operation for median lithotomy was done, which very much relieved him, and thus he was saved the torture of many catheterizations A drainage tube was inserted; this had to be frequently removed and freed from the deposits of phosphates and retention of mucus. The urine was excessively ammoniacal and fœtid. But the patient survived only a few days. Autopsy; cirrhotic, gouty liver and kidneys, cystitis of aggravated and obstinate form, enlarged prostate, etc.

The speaker thought he was warranted in making the median incision in the case, he reported, and should do so again under similar circumstances.

Dr. T. C. Parkes supported Dr. Axford in what he had done in the case. He then briefly alluded to two cases of a similar nature,—relieved by this method,—that remained so for six months. Dr. Parkes then gave an interesting verbal report of a case, and also exhibited the specimen, which consisted of papillomatous, multilocular cysts of both ovaries. The cysts had ruptured, and discharged their contents into the peritoneal sac. The history of enlargement embraces a period of only six months. The patient stated she never had an illness previous to the commencement of the abdominal enlargement. She has emaciated rapidly and to great extent. The patient measured 44 inches in circumference at height of umbilicus. The greatest difficulty in the case was the differentiation between ascites and ovarian cysts. There was no œdema, nor had there ever been, of the lower extremities. Percussion gave dullness in all positions—lying, standing, or sitting, the only place of resonance being in the epigastrium. Fluctuation was very distinct on the slightest touch in all directions. Pelvic examination revealed that fluctuation was not to be felt in the pelvis. By this means, was also determined the presence of a small growth about the size of the closed fist on the right side. The uterus was displaced forward, close behind the pubes; *cul de sac* of Douglas filled with foreign body. *Diagnosis:*—Ovarian tumor; ruptured sac; contents emptying into peritoneal sac. *Operation:*—22 quarts of fluid were evacuated from the abdomen and tumors; fluid of a dark amber color. No large amount of lymph was precipitated in it after standing. Both ovaries were found diseased, there being present in the left side a ruptured papillomatous tumor. It still contained some fluid, which could not be pressed out at the site of papillary growth. The right one had not ruptured, but was bound down deeply in the pelvis by adhesion. The pelvis was filled by these tumors and their adhesions. After much trouble, the tumors were enucleated and removed, which were, at this point, shown to the members. The operation was done recently, and during the first four days the temperature of the patient had not reached 100° F. She was strong and cheerful, and she was doing well in every respect, but was not yet out of danger. Bleeding was quite free immediately after the separation of the adhesions, but soon ceased altogether under pressure with dry sponges. A drainage-tube was left in the lower end of the incision down to the floor of the pelvis, anticipating free discharge from such an extensive raw surface. The drain off was very free through it for two days. The sigmoid flexure of the colon was adherent for two inches to one of the cysts. For the past two days the temperature was normal.

Dr. A. R. Jackson remarked: The distention by the fluids, in this case, prevented the intestines from rising to the highest point in the abdomen; and it required, he thought, great skill on the part of the operator to differentiate such a case from one of ascites, hydronephrosis, or ovarian tumor, although before proceeding to operate, an examination of the fluid would have determined it to be ovarian or otherwise. Drysdale has made 2,500 examinations, and, with but one or two exceptions (the speaker thinks), has succeeded in finding the characteristic pathognomonic cell in ovarian cystic fluid. L. H. M.

